# Direct Electron Transfer of Enzymes Facilitated by Cytochromes

**DOI:** 10.1002/celc.201801256

**Published:** 2018-12-13

**Authors:** Su Ma, Roland Ludwig

**Affiliations:** ^1^ Biocatalysis and Biosensing Laboratory Department of Food Science and Technology BOKU – University of Natural Resources and Life Sciences Muthgasse 18 1190 Vienna Austria

**Keywords:** cytochrome, direct electron transfer, direct electrochemistry, flavin, flavocytochrome, haem, molybdenum, multi-cofactor enzyme, pyrrolinoquinoline quinone, iron-sulfur cluster

## Abstract

The direct electron transfer (DET) of enzymes has been utilized to develop biosensors and enzymatic biofuel cells on micro‐ and nanostructured electrodes. Whereas some enzymes exhibit direct electron transfer between their active‐site cofactor and an electrode, other oxidoreductases depend on acquired cytochrome domains or cytochrome subunits as built‐in redox mediators. The physiological function of these cytochromes is to transfer electrons between the active‐site cofactor and a redox partner protein. The exchange of the natural electron acceptor/donor by an electrode has been demonstrated for several cytochrome carrying oxidoreductases. These multi‐cofactor enzymes have been applied in third generation biosensors to detect glucose, lactate, and other analytes. This review investigates and classifies oxidoreductases with a cytochrome domain, enzyme complexes with a cytochrome subunit, and covers designed cytochrome fusion enzymes. The structurally and electrochemically best characterized proponents from each enzyme class carrying a cytochrome, that is, flavoenzymes, quinoenzymes, molybdenum‐cofactor enzymes, iron‐sulfur cluster enzymes, and multi‐haem enzymes, are featured, and their biochemical, kinetic, and electrochemical properties are compared. The cytochromes molecular and functional properties as well as their contribution to the interdomain electron transfer (IET, between active‐site and cytochrome) and DET (between cytochrome and electrode) with regard to the achieved current density is discussed. Protein design strategies for cytochrome‐fused enzymes are reviewed and the limiting factors as well as strategies to overcome them are outlined.

## 1 Direct Electron Transfer Capable Enzymes

The direct electron transfer (DET) or direct electrochemistry between an oxidoreductase and an electrode is the initiating step of the oxidative half‐reaction in oxidases and dehydrogenases and, vice versa, the initial step of the reductive half‐reaction in reductases. DET is therefore the prior condition for catalytic turnover and direct bioelectrocatalysis. In direct bioelectrocatalysis an anode substitutes soluble or membrane‐bound electron acceptors for dehydrogenases and oxidases, whereas a cathode replaces the electron donor for reductases. DET‐capable oxidoreductases are applied as anode biocatalysts (e. g. cellobiose dehydrogenase or sulfite dehydrogenase) or cathode biocatalysts (e. g. laccase or bilirubin oxidase) in third generation biosensors, mediator‐free biofuel cells and bioelectrosynthesis. Since DET was discovered for cytochrome *c* in 1973 by Yeh and Kuwana as well as Eddows and Hill,^1^ great efforts have been made to discover DET‐capable enzymes. A flavocytochrome *b*_2_‐based biosensor without soluble redox mediator was developed in 1980, which for the first time showed that a cytochrome domain can work as an IET unit in oxidoreductases.^2^

Multi‐cofactor enzymes carry a cytochrome domain, or enzyme complexes with a cytochrome subunit, but also oxidoreductases with a copper cofactor (laccase, bilirubin oxidase, galactose oxidase and ascorbate oxidase),^3^ with a haem cofactor (cytochrome *c* peroxidase, horseradish peroxidase, lignin peroxidase and manganese peroxidase)^4^ or with an iron‐sulfur cluster (NiFe hydrogenase and FeFe hydrogenase)^5^ have been applied in DET‐based electrodes. Important features of enzymes in DET applications are a high catalytic activity and stability in its electrode‐bound form together with a beneficial orientation onto the electrode that results in a high DET rate. Efficient DET in which the electron transfer rate does not limit the catalytic turnover rate is rarely observed but involving cytochromes as build‐in redox mediators often results in a DET rate sufficient for the application. Cytochrome *c* can bridge the reaction of oxidoreductases to electrodes, which was demonstrated for cellobiose dehydrogenase, bilirubin oxidase and sulfite oxidase.^6^ Cytochromes have been evolved as reversible shuttles for electron transfer pathways over a long time, e. g. the electron transfer chain in the mitochondrion, which appeared 1.7–2×10^6^ years ago.

The performance of third generation biosensors and biofuel cells critically depends on the DET rate, which can become the rate‐limiting step for catalysis. An increased DET goes hand‐in‐hand with higher turnover numbers, higher specific currents, higher sensitivities and lower detection limits. It also helps to lower the polarization potential of third generation biosensors, which decreases the rate of interfering oxidation processes at the working electrode and thereby increases the selectivity of the biosensor. The optimization of biocatalysts and their molecular, catalytic or electron transferring properties becomes simpler due to the rapid development of protein engineering techniques.

Recent protein engineering efforts mimic naturally evolved electron transfer proteins and enzymes by fusing a cytochrome domain to glucose dehydrogenase. A good interaction of the cytochrome with the active‐site cofactor is important to obtain high IET and DET rates and consequently a high current density for glucose biosensors. To evaluate the potential of enzymes featuring an electron transferring cytochrome for third generation biosensors and biofuel cells, this review presents and classifies multi‐cofactor enzymes, evaluates the molecular requisites for DET and investigates the potential of engineered enzymes with a fused cytochrome domain for DET.

## 2 History, Classification and Properties of Cytochromes

The name cytochrome was introduced in 1925 by David Keilin^7^ based on work from Charles Alexander Mac Munn^8^ who found the typical cytochrome spectra in muscles, tissues and organs of invertebrate and vertebrate animals. The absorption bands are different from haemoglobin, because of the distal amino acid ligand which is missing in the oxygen transporter protein. Mac Munn found that the α‐ and β‐bands are only visible in the reduced state of the cytochrome and from the distinct oxidation states he deduced a respiratory function. As predicted, cytochromes turned out to be components of the mitochondrial electron transfer chain and work in many other functions as specific electron acceptors or electron donors. Cytochromes evolved specific protein interfaces to complement and recognize their redox partners and perform fast electron transfer.

The detection and identification of cytochromes is supported by their distinctive, redox‐sensitive spectra. Individual cytochromes are resolved on the basis of their lowest energy absorption peak, the α‐band.^9^ Based on their different absorption bands, early discovered cytochromes were denoted *a*, *b and c*, which still forms the basis of the current classification into six classes: *a*, *b*, *c*, *d*, *f* and *o*. These cytochromes differ in the chemical substituents of the haem tetrapyrrole ring, its linkage to the protein, or both. Two different nomenclatures are used to denote subclasses following either function (e. g. cytochrome *c_1_*) or giving the α‐band absorption maximum (cytochrome *c_550_*).^10^ Another classification based on characteristic sequence motifs, which result in different properties, divides cytochrome *c* into four subclasses (I–IV).^11^

In multi‐cofactor enzymes two cytochrome types are found: cytochrome *b* and *c*. The difference lies in the covalent attachment of the protoporphyrin IX ring in *c*‐type cytochromes via two thioether bonds between its vinyl groups and the two cysteine residues from the typical cytochrome *c* pentapeptide CXXCH motif, whereas haem *b* is not covalently bound. The protein's binding pocket also determines the haem's function including electron transfer, oxygen transport, catalysis and others. The protein environment modifies the haem reactivity by the axial iron ligands, the haem burial in the protein and its solvent accessibility, and the arrangement of polar or charged amino acid side chains around the haem.

In cytochromes two axial iron ligands prevent the access of small molecule ligands or substrates and determine its function as an electron storage and electron transfer protein. In cytochrome *b*, the proximal and distal ligands are usually histidine imidazole groups (His‐His), whereas in cytochrome *c*, one His imidazole is typically replaced by a Met sulfur group (His‐Met). It is a convention, that the His of the CXXCH motif is referred to as the proximal ligand and the other side of the haem as the distal side. A strong ligation of the iron results in a low‐spin state and typically the iron center in cytochromes alternates between an oxidized Fe(III) low‐spin state with a single unpaired electron and a formal charge of +1 and a reduced Fe(II) low‐spin form with no unpaired electrons and a net charge of zero. The change between the reduced and oxidized state is facilitated by the iron remaining in the low‐spin state.^12^ Few high‐spin cytochromes such cytochrome *c*’ are known. A weak or no axial coordination of the iron results in the high spin‐state and correspondingly a low redox potential.^13^

The cofactors of metalloproteins exhibit potentials between −700 to +780 mV vs. SHE. For cytochromes redox potentials between −400 and +400 mv vs. SHE are reported (typically above of iron‐sulfur clusters and below cupredoxins), which define their biochemical functions. The redox potential of the haem cofactor is modulated by the stabilization of the buried haem by the protein matrix, modulation by polar and ionized side chains and backbone dipoles; the axial ligands, ligand geometry and first‐ and second coordination sphere of the iron; haem type and covalent bond to the haem, solvent accessibility, ionic composition and pH of the solvent, electrostatic interactions with the environment, and temperature.^14^ The typically higher redox potentials of *c*‐type cytochromes over *b*‐type cytochromes mainly arise from the π‐electron‐acceptor character of the thioether sulfur atom of the distal Met, which stabilizes the ferrous over the ferric state.10a

**Figure 1:**
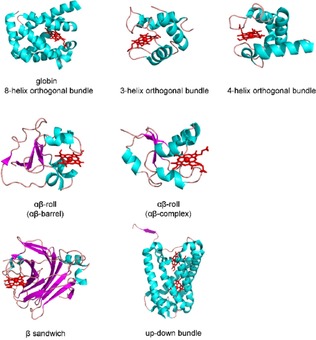
Cytochrome folds. Globin (8‐helix orthogonal bundle, flavohemoglobin 4G1V); 3‐helix orthogonal bundle (*p*‐cresol methylhydroxylase 1DII); 4‐helix orthogonal bundle (cytochrome *cd_1_* 1H9X); αβ‐barrel (flavocytochrome *b_2_* 1FCB); αβ‐complex (sulfite oxidase 1SOX); β‐sandwich (cellobiose dehydrogenase 4QI7); up‐down bundle (nitrate reductase 1Q16).

Cytochromes are electron storing and transferring haemoproteins, which were evolved from a variety of existing haemoprotein folds. This results in the structural diversity of cytochrome protein‐fold families (Figure 1) such as: globin (8‐helix orthogonal bundle, e. g. cytochrome *c_551_*, cytochrome *b* from flavohemoglobin), orthogonal bundle (3 helices, e. g. cytochrome *c_6_*, cytochrome *c* from *p*‐cresol methylhydroxylase; 4 helices, e. g. cytochrome *c* from cytochrome *cd_1_*), αβ‐roll (αβ‐barrel, e. g. cytochrome *b_5_*, cytochrome *b* from flavocytochrome *b_2_*; αβ‐complex, e. g. cytochrome *b* from sulfite oxidase), β‐sandwich (cytochrome *f*, cytochrome *b* from CDH) up‐down bundle (e. g. cytochrome *b_562_*, cytochrome *b* from *E. coli* nitrate reductase A). Therefore, cytochromes vary in molecular mass, surface geometry, isoelectric point and surface charge distribution, but also show variations in the haem environment, ligation geometry and therefore the redox potential.

The best‐studied cytochrome is cytochrome *c*. It is also the model protein for DET studies in electrochemistry. Many electrode materials and surface modifications have been applied to promote DET of cytochrome *c*. Also, the immobilization method shows an influence on DET, especially the orientation of the cytochrome on the electrode. Oriented immobilization has been employed to optimize the haem‐to‐electrode distance and thereby the electronic coupling of the haem to the electrode.^15^ The most specific and defined experimental conditions are achieved with metal electrodes coated with self‐assembled monolayers (SAM) of alkanethiols, which can feature a variety of different head groups to generate different apolar, polar, or charged environments for the cytochrome. But also different carbon electrodes and carbon based materials such as carbon nanotubes, derivatized reduced graphene oxide, boron‐doped diamond electrodes or porous silicon materials were used.10a

## 3 Multi‐Cofactor Oxidoreductases Carrying a Cytochrome

**Figure 2:**
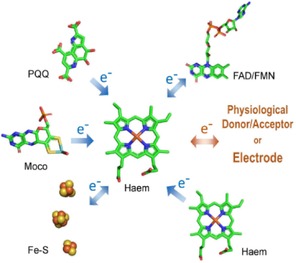
Scheme of interacting cofactors in multi‐cofactor oxidoreductases with a distinct cytochrome domain or multi‐cofactor enzyme complexes with a cytochrome subunit. The active‐site cofactors and cofactors neighboring the haem can be: 1) flavin adenine mononucleotide (FMN) or dinucleotide (FAD), 2) pyrroloquinoline quinone (PQQ), 3) a Mo atom bound by a molybdopterin cofactor (Moco), 4) iron‐sulfur cluster of different types (Fe_2_S_2_, Fe_3_S_3_, Fe_4_S_4_), and 5) a catalytic haem (Haem). In oxidative processes, the electrons are transferred from the catalytic cofactor to the electron transferring cytochrome, which then transfers the electrons to the terminal, physiological electron acceptor protein or the electrode.

Searching the EC classification of enzymes, we found 39 sub‐sub classes of an enzyme featuring a cytochrome domain or a cytochrome subunit that is responsible for the intermolecular electron transfer to or from an electron donor or acceptor protein (Table S1). They belong to EC class 1 (oxidoreductases) and are multi‐cofactor enzymes containing flavins, porolinoquinoline quinone, different haem‐types, iron‐sulfur clusters or molybdenum as prosthetic groups and a cytochrome domain or cytochrome subunit as electron transferring moiety. Here, the differentiation into cytochrome domains and subunits distinguishes single chain multi‐cofactor enzymes featuring the cytochrome as a domain of the enzyme and multi‐cofactor enzyme complexes with a co‐assembled cytochrome subunit. About half (20) of the EC sub‐sub classes feature membrane‐associated multi‐cofactor enzymes that participate in electron transport chains. The majority of membrane‐associated enzymes are complexes (17) consisting of at least two subunits, whereas only 3 single‐chain membrane‐associated enzymes were found. The other half of sub‐sub classes (19) feature soluble enzymes, which interact with soluble or membrane‐associated electron transfer proteins. Most of the soluble enzymes (16) are single polypeptide chains and only 3 are enzyme complexes. An overview on the different active‐site cofactors in cytochrome carrying multi‐cofactor enzymes is presented in Figure 2.

The found multi‐cofactor oxidoreductases can be classified by their active‐site cofactor, by the type and number of the cytochrome, or by their catalytic activity or physiological function. Since the 39 found examples have very diverse activities and are located in various intra‐ and extracellular places, the enzymatic activity and physiological function is not a suitable descriptor for the classification of the enzymes in this review. A systematic approach, justified by their different functions and redox properties, is the classification according to their (1) active‐site cofactor or cofactor closest to the cytochrome and (2) the type and number of cytochromes. The catalytic activity and physiological function of enzymes in the same class and even subclass can differ strongly. By sorting enzymes from the 39 EC sub‐sub classes according to these criteria following classes are obtained: (1) flavoenzymes carrying a cytochrome (flavocytochromes), (2) PQQ‐enzymes carrying a cytochrome, (3) molybdenum cofactor enzymes carrying a cytochrome, (4) Iron‐sulfur enzymes carrying a cytochrome, and (5) multihaem enzymes carrying a cytochrome. Prominent and electrochemically characterized enzymes of each class are listed and classified in the following five subsections.

### 3.1 Flavoenzymes Carrying a Cytochrome

**Figure 3:**
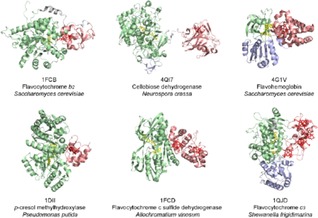
Multi‐cofactor flavoenzymes sorted by their cytochromes. Subclass 1 contains soluble flavocytochromes with a mobile *b‐*type cytochrome domain (1FCB, 4QI7, 4G1V). Subclass 2 features a membrane‐associated enzyme complex with a *c*‐type cytochrome (1DII). Subclass 3 features multiple haems of either membrane‐associated (1FCD, 2 haems) or soluble (1QJD, 4 haems) flavocytochromes.

Flavoenzymes with an attached cytochrome domain catalyze the dehydrogenation, mono‐oxygenation or deoxygenation of substrates. By sorting them according to their cytochrome type, three subclasses can be separated. The first subclass consists of soluble (intra‐ or extracellular) flavoenzymes with a *b*‐type cytochrome: L‐lactate dehydrogenase (flavocytochrome *b_2_*), EC 1.1.2.3; cellobiose dehydrogenase, EC 1.1.99.18; and nitric oxide dioxygenase (flavohemoglobin), EC 1.14.12.17. The second subclass contains membrane‐associated enzyme complexes with a *c*‐type cytochrome: gluconate 2‐dehydrogenase, EC 1.1.99.3; *p*‐cresol methylhydroxylase, EC 1.17.99.1; and spermidine dehydrogenase, EC 1.5.99.6. The third subclass contains flavocytochromes with multiple *c*‐type haems: sulfide‐cytochrome‐*c* reductase (flavocytochrome c sulfide dehydrogenase), EC 1.8.2.3; NADPH oxidase, EC 1.6.3.1; fructose dehydrogenase, EC 1.1.99.11; and fumarate reductase (flavocytochrome *c_3_*), EC 1.3.1.6. Available structures of these multi‐cofactor flavoenzymes are given in Figure 3.

Cellobiose dehydrogenase and L‐lactate dehydrogenase are intensively studied biocatalysts for biosensors and biofuel cells.^16^ Also membrane‐associated multi‐cofactor enzyme complexes were applied for biosensors. A D‐fructose biosensor was successfully developed and optimized based on the DET of fructose dehydrogenase^17^ and a biosensor for gluconic acid was constructed based on D‐gluconate 2‐dehydrogenase adsorbed on bare and thiol‐modified fold electrodes.^18^

### 3.2 PQQ Enzymes Carrying a Cytochrome

**Figure 4:**
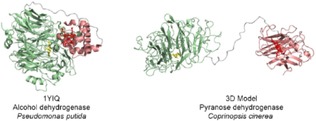
Two soluble PQQ‐enzymes with flexible cytochrome domains. The intracellular alcohol dehydrogenase features a *c*‐type cytochrome (1YIQ), whereas in the model of the extracellular pyranose dehydrogenase (open conformation) a *b*‐type cytochrome is found.

Similar to the flavocytochromes, three cytochrome subclasses can be assigned in PQQ‐dependent multi‐cofactor enzymes: (1) carrying a *b‐*type cytochrome: pyranose dehydrogenase, EC 1.1.2.B5; (2) carrying a *c*‐type cytochrome: lupanine hydroxylase (EC 1.17.2.2), lactate dehydrogenase (EC 1.1.5.B3), polyvinyl alcohol dehydrogenase (EC 1.1.2.6), 1‐butanol dehydrogenase (EC 1.1.2.9), type II alcohol dehydrogenases (EC 1.1.9.1) and a novel pyruvate dehydrogenase from *Gluconobacter* sp.; (3) carrying multiple haem *b/c* moieties: type III alcohol dehydrogenase, EC 1.1.5.5 and aldehyde dehydrogenase, EC 1.2.5.2. Subclasses 1 and 2 are composed of monomeric enzymes with a distinct domain for each cofactor and soluble. In subclass 3, membrane‐associated type III alcohol dehydrogenase and aldehyde dehydrogenase heterodimers feature an additional 50 kDa subunit containing three haem *c* moieties.^19^ Only one structure of a type II alcohol dehydrogenase with a *c*‐type cytochrome has been elucidated so far (1YIQ), it is presented together with a structural model for a promising enzyme from subclass 1: pyranose dehydrogenase (Figure 4).

Soluble PQQ‐dependent pyranose dehydrogenase is capable of DET on glassy carbon electrodes.^20^ Biosensors and bioanodes based on PQQ‐alcohol dehydrogenase or PQQ‐aldehyde dehydrogenase were studied already 20 years ago, by both mediated electron transfer (MET) and DET.^19^,^21^ Recently, newly discovered PQQ‐dependent pyruvate and lactate dehydrogenases from acetic acid bacteria were demonstrated to exhibit DET.^22^

### 3.3 Molybdenum Cofactor Enzymes Carrying a Cytochrome

**Figure 5:**
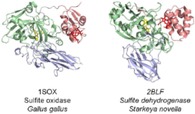
The soluble sulfite oxidase carries a mobile cytochrome *b* (1SOX), whereas the membrane‐associated sulfite dehydrogenase (2BLF) features a fixed cytochrome *c* subunit.

Two enzyme classes contain a molybdenum atom complexed by a molybdopterin molecule in combination with a cytochrome. This molybdenum cofactor is often abbreviated as Moco. Sulfite oxidase (EC 1.8.3.1) carrying a mobile *b*‐type cytochrome domain and is found in vertebrates, while sulfite dehydrogenase (EC 1.8.2.1) carries a fixed *c*‐type cytochrome subunit and is found in bacteria. The proposed catalytic mechanism of sulfite oxidase and sulfite dehydrogenase involves two intramolecular one‐electron transfer steps from the molybdenum cofactor to a *b*‐ or c‐type cytochrome, respectively.^23^ In the vertebrate sulfite oxidases the cytochrome and molybdenum domains are linked by a flexible loop and catalysis requires a repositioning of the cytochrome domain to allow electron transfer. In bacterial sulfite dehydrogenase the haem and molybdenum cofactors are located on separate subunits and the position of these subunits does not change during catalysis (Figure 5).^24^

Direct electrochemistry using different electrode materials was observed for sulfite dehydrogenase23b,^24^, ^25^ and sulfite oxidase.^26^ A DET based sulfite/oxygen biofuel cell was reported using human sulfite oxidase and bilirubin oxidase on modified gold electrodes.^27^ Power densities of 1 and 8 μW cm^−2^ were achieved at different polarization potentials. A third‐generation sulfite biosensor was developed based on human sulfite oxidase on indium tin oxide, which exhibited a sensitivity of 35 nA mM^−1^ and a linear range between 0.5–20 μM.^28^ Another sulfite biosensor was developed using bacterial sulfite dehydrogenase, cytochrome *c* as mediator and a self‐assembled monolayer of 11‐mercaptoundecanol cast on a gold electrode.^29^

### 3.4 Iron‐Sulfur Enzymes Carrying a Cytochrome

**Figure 6:**
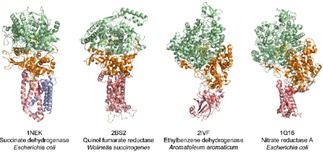
Iron–sulfur cluster enzymes featuring a catalytic FAD cofactor and a cytochrome *b* subunit (1NEK, one *b*‐type haem; 2BS2, two *b*‐type haems) or a catalytic molybdenum cofactor and a cytochrome *b* subunit (2IVF, one *b*‐type haem; 1Q16, two *b*‐type haems).

Enzyme complexes containing an iron‐sulfur cluster and a cytochrome are intracellularly located either in the periplasm or are membrane bound. They commonly contain also other cofactors, such as flavin, molybdenum, haem or nickel. These active‐site cofactors catalyze oxidation, reduction, oxygenation or hydroxylation reactions. The iron‐sulfur clusters function as an electron transferring unit in these mostly membrane‐associated enzyme complexes. Based on the haem types, iron‐sulfur enzymes can be classified into three subgroups containing: (1) a single or two *b*‐type cytochrome(s): succinate dehydrogenase, EC 1.3.5.1; fumarate reductase, EC 1.3.5.4; molybdenum: nitrate reductases, EC 1.7.5.1; dimethyl sulfide/cytochrome *c_2_* reductase, EC 1.8.2.4; ethylbenzene hydroxylase, EC 1.17.99.2; chlorate reductase, EC 1.97.1.1; selenite reductase, EC 1.97.1.9; CoB‐CoM heterodisulfide reductase (coenzyme), EC 1.8.98.1; hydrogen/quinone oxidoreductase (Ni), EC 1.12.5.1; (2) two *c*‐type cytochromes: nitrate reductase, EC 1.9.6.1 and (3) a sirohaem/nitrite reductase, EC 1.7.1.15; assimilatory sulfite reductase, EC 1.8.1.2 (Figure 6).

Over the last two decades, significant efforts to develop nitrate biosensors have been directed to immobilization within or on conducting and non‐conducting polymers.^30^ Mediatorless whole‐cell sensors have been developed mainly using *E. coli* or denitrifying bacteria. Direct electrochemistry of NarGHI from *E. coli* was reported using a pyrolytic graphite “edge” rotating electrode.^31^ Catalytic cyclic voltammetry was performed in the presence of nitrate and resulted a catalytic current of 0.9 μA at a potential of −400 mV vs. SHE.

### 3.5 Multihaem Enzymes Carrying a Cytochrome

**Figure 7:**
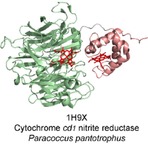
The structure of the soluble cytochrome *cd_1_* nitrite reductase (1H9X) featuring a mobile cytochrome *c* domain and a catalytic haem *d_1_*.

Several multihaem oxidoreductases that act on nitrogenous compounds are listed in enzyme sub‐sub class E.C. 1.7.2. carry a cytochrome as acceptor. These contain either: (1) the same haem‐type, e. g. hydroxylamine dehydrogenase EC 1.7.2.6 featuring seven *c*‐type haems), or (2) different haem‐types, e. g. cytochrome *cd*_1_ EC 1.7.2.1 with a haem *c* and a haem *d_1_*, Figure 7).

Cytochrome *cd_1_* has been used for the determination of nitrites using cytochrome *c_552_* as a redox mediator.^32^ The sensitivity of the nitrite sensor was 2.49±0.08 A mol^−1^ cm^2^ μM^−1^.

## 4 Structural Properties of Multi‐Cofactor Enzymes Carrying a Cytochrome

Multi‐cofactor enzymes with a known protein structure are listed in Table 1. The structural data were used to investigate the fold of their cytochrome, the axial haem ligands, and to calculate various molecular properties of the listed cytochromes. It has to be noted that not all of these enzymes have a verified DET. Enzyme complexes (which are mostly membrane associated) feature an immobile cytochrome subunit, which can be well accessed by other redox proteins in the electron transfer chain, but in some cases might not interact with the electrode surface in an orientation that is favorable for DET. Contrary, the soluble, intra‐ or extracellular multi‐cofactor enzymes feature a cytochrome domain connected by a linker to the catalytic domain. These enzymes are typically smaller than the enzyme complexes and have a higher mobility of their cytochrome domain All enzymes in Table 1 feature either *b*‐ or *c*‐type cytochromes in various protein folds.

**Table 1:** Multi‐cofactor enzyme structures according to their classification by cofactors and their most important structural and functional properties.

EC number systematic enzyme name	Common enzyme name Source PDB structure ID	Cellular location	Cytochrome fold CATH‐classification	Cytochrome attachment	Axial haem ligands	Catalytic or neighboring cofactor	Cofactor edge‐to‐edge distance [nm]	Cytochrome termini, type and length	Theoretical pI of cytochrome	Cytochrome volume [nm^3^]^37^	Interface area [nm^2^]	Haem redox potential [mV vs. SHE]	Ref.
Class 1: Flavoenzymes carrying a cytochrome													
EC 1.1.2.3 L‐lactate dehydrogenase	Flavocytochrome *b_2_ Saccharomyces cerevisiae* 1FCB	soluble	αβ‐roll (αβ‐barrel)	domain	1 *b*‐type His/His	FMN	0.53	E1‐G86 domain 86 aa	7.1	11.38	10.93	202 (pH 7.0)	[38]
EC 1.1.99.18 cellobiose dehydrogenase	*Neurospora crassa* 4QI7	soluble	β‐sandwich	domain	*1 b‐*type His/Met	FAD	3.87	E1‐G213 domain 213 aa	5.46	26.96	open conformation	99 (pH 6.0) 93 (pH 7.5)	[39]
	*Myriococcum thermophilum* 4QI6	soluble	β‐sandwich	domain	*1 b‐*type His/Met	FAD	0.86	N1‐D208 domain 208 aa	4.07	26.1	8.64	151 (pH 5.5)	[39b,40]
EC 1.14.12.17 nitric oxide dioxygenase	Flavohemoglobin *Saccharomyces cerevisiae* 4G1V	soluble	globin (8‐helix orthogonal bundle)	domain	1 *b*‐type His	FAD	0.61	M1‐A144 domain 144 aa	6.51	19.49	11.05	34 (pH 7.4) (*E.coli*)	[41]
EC 1.17.99.1 4‐methylphenol dehydrogenase	*p*‐Cresol methylhydroxylase *Pseudomonas putida* 1DII	membrane associated	3‐helix orthogonal bundle	subunit	1 *c*‐type His/Met	FAD	0.76	D601‐P674 subunit 74 aa	5.14	9.32	9.7	250 (pH 7.0)	[42]
EC 1.8.2.3 sulfide‐cytochrome *c* reductase	Flavocytochrome *c* sulfide dehydrogenase *Chromatium vinosum* 1FCD	membrane associated	4‐helix orthogonal bundle	subunit	2 *c*‐type His/Met	FAD	0.98	E1‐Q174 subunit 174 aa	5.23	22.28	14.16	220 (pH 7.0)	[43]
EC 1.3.1.6 fumarate reductase	Flavocytochrome *c_3_ Shewanella frigidimarina* 1QJD	soluble	3‐helix orthogonal bundle	domain	4 *c*‐type His/His	FAD	0.47	A1‐R100 domain 100 aa	5.25	12.36	9.36	−102–146, −196–238 (pH 7.0)	[44]
Class 2: PQQ‐enzymes carrying a cytochrome													
EC 1.1.9.1 alcohol dehydrogenase	Alcohol dehydrogenase *Pseudomonas putida* 1YIQ	soluble	4‐helix orthogonal bundle	domain	1 *c*‐type His/Met	PQQ	1.22	D591‐S684 domain 94 aa	6.13	11.91	19.19	185 (pH 7.0) 188 (pH 8.0)	[45]
Class 3: Molybdenum cofactor enzymes carrying a cytochrome													
EC 1.8.3.1 sulfite oxidase	Sulfite oxidase *Gallus* 1SOX	soluble	αβ‐roll (αβ‐complex)	domain	1 *b*‐type His/His	molybdenum	2.02	A3‐P84 domain 82 aa	4.86	10.67	6.47	68 (pH 7.0)	[46]
EC 1.8.2.1 sulfite dehydrogenase	Sulfite dehydrogenase *Starkeya novella* 2BLF	membrane associated	4‐helix orthogonal bundle	subunit	1 *c*‐type His/Met	molybdenum	0.7	A501‐Y581 subunit 81 aa	4.67	9.99	8	242 (pH 8.0)	[24,47]
Class 4: Iron‐sulfur enzymes carrying a cytochrome													
EC 1.3.5.1 succinate dehydrogenase	Succinate dehydrogenase *Escherichia coli* 1NEK	membrane associated	up‐down bundle	subunit	1 *b*‐type His/His	FAD, Fe−S cluster	0.83	M1‐W129 & S3‐V115 subunit 242 aa	9.56	32.05	16.78	36 (pH 7.0)	[48]
EC 1.3.5.4 fumarate reductase	Quinol/fumarate reductase *Wolinella succinogenes* 2BS2	membrane associated	up‐down bundle	subunit	2 *b*‐type His/His	FAD, Fe−S clustur	0.83	M1‐E255 subunit 255 aa	9.34	35.05	15.79	haem *b*_H_ ‐9 h *b*_L_ −152 (pH 7.0)	[49]
EC 1.17.99.2 ethylbenzene hydroxylase	Ethylbenzene dehydrogenase *Aromatoleum aromaticum* 2IVF	membrane associated	up‐down bundle	subunit	1 *b*‐type Lys/Met	molybdenum, Fe−S clustur	0.65	M1‐K214 subunit 214 aa	9.18	27.32	9.61	256(pH 7.0)	[50]
EC 1.7.5.1 nitrate reductase	Nitrate reductase A *Escherichia coli* 1Q16	membrane associated	up‐down bundle	subunit	2 *b*‐type His/His	molybdenum, Fe−S clustur	0.89	Q2‐H225 subunit 224 aa	9.77	29.49	24.67	haem *b*_H_ 120 haem *b*_L_ 20 (pH 7.0)	[51]
Class 5: Multihaem enzymes carrying a cytochrome													
EC 1.7.2.1 nitrite reductase	Cytochrome *cd_1_ Paracoccus pantotrophus* 1H9X	soluble	4‐helix orthogonal bundle	domain	1 *c*‐type His/Met	*d*_1_‐type heme	0.95	L49‐D130 domain 82 aa	4.47	10.88	8.06	249 (pH 6.6)	[52]

The observed cytochrome *b* folds are: 8‐helix orthogonal bundle (globin), up‐down bundle, β‐sandwich, and the αβ‐barrel and αβ‐complex (αβ‐roll). The observed cytochrome *c* folds are: 3‐helix orthogonal bundle, 4‐helix orthogonal bundle and αβ‐complex. The cytochromes vary in size, with the 3‐helix orthogonal bundle (40–74 aa) being the smallest, the 4‐helix orthogonal (80–94 aa), αβ‐roll (82–86 aa) and 8‐helix orthogonal bundle (144 amino acids) in the median range, and the β‐sandwich (208–213 aa) and the up‐down bundle (214–255 aa) being about twice as big. The volume of the cytochrome domains vary also according to the number of accommodated amino acids and is typically smaller for the single‐haem cytochromes (10–12 nm^3^) and bigger for the multi‐haem cytochromes (22–32 nm^3^). Exceptions are the big single‐haem β‐sandwich fold (∼26 nm^3^) and the structurally very compact cytochrome domain of flavocytochrome *c_3_* (12 nm^3^), which contains 4 haem cofactors.

The ligation of the haem iron in the proximal and distal position is most often typical and shows His/His ligation for cytochrome *b* and His/Met ligation for cytochrome *c*. Exceptions from this rule are found in *b*‐type cytochromes: cellobiose dehydrogenase with a His/Met ligation, flavohaemoglobin with only a proximal His ligand and ethylbenzene dehydrogenase with a Lys/Met; and *c*‐type cytochrome: fumarate reductase with a His/His.

The observed edge‐to‐edge distances between the haem and the closest cofactor in the closed conformation/in the enzyme complex are between 0.47 and 1.22 nm, the median distance being 0.76 nm. The interface between the catalytic domain or subunit and the cytochrome domain or subunit has an area between 8 and 24 nm^2^ (the interface area is the combined area of both interacting surfaces) with a tendency of mobile cytochrome domains having a smaller interface and fixed subunits having a bigger. Typical protein‐protein interfaces show an interface size distribution between 2 and 28 nm^2^, with 8 nm^2^ being the median size. The strength of the interaction is enhanced by steric surface complementarity, opposite charge pairs and hydrophobic pairs of amino acids.^33^ Therefore, not only the interaction surface area, but also the match of opposing amino acid residues defines how strong the cytochrome is bound. For a cytochrome domain which is attached to the catalytic domain of the molecule, e. g. cellobiose dehydrogenase, the domain interaction is fine‐tuned. A too weak binding makes a close conformation of both cofactors unlikely and reduce the IET, a too strong binding will prevent the dissociation of the cytochrome domain and can reduce DET. Theoretical investigations based on molecular dynamics (MD) simulations of sulfite oxidase showed that the high mobility of the cytochrome domain was enabled by the flexible linker region^34^ and higher ionic strength weakened the interaction with the enzyme, which increased intramolecular domain motions.^35^ When sulfite oxidase is bound to an electrode, the conformational changes occur at a lower rate than in solution, which decreases the activity.^36^

## 5 Functional Properties of Multi‐Cofactor Enzymes Carrying a Cytochrome

The cytochrome domain acts as an electron acceptor in dehydrogenases/oxidases and as an electron donor in reductases. Correspondingly, the redox potential of the cytochrome in the investigated dehydrogenases and oxidases is 34 to 494 mV vs. SHE to function as an electron acceptor of the catalytic cofactor, whereas in reductases the redox potential is between −238 to 20 mV vs. SHE. Details of multi‐cofactor enzymes with a demonstrated DET (flavocytochromes, PQQ‐enzymes, molybdenum enzymes, iron‐sulfur enzymes and fusion enzymes) are listed in Table 2. Two enzymes with an unusual haem iron ligation (flavohaemoglobin with a proximal His and ethylbenzene dehydrogenase with a Lys/Met) do not exhibit DET.

**Table 2:** Electrochemical and kinetic data of multi‐cofactor enzymes with reported DET.

Enzyme name EC number		Redox potential [mV vs. SHE]	Redox potential difference [mV]	TN of active‐site [s^−1^]	IET rate [s^−1^]	DET rate according to Ref. (or calculated) [s^−1^]	Electrode architecture	Sensor analyte or biofuel cell substrate	Sensor/fuel cell architecture	Current density [nA mm^−2^]	Sensitivity [nA mM^−1^ mm^−2^]	Ref.
Class 1: Flavoenzymes carrying a cytochrome												
l‐lactate dehydrogenase EC 1.1.2.3		FMN 115, haem *b* 202 (pH 7.0)	87 (pH 7.0)	214 using L‐lactate and ferricyanide (pH 7.0)	90 (pH 7.0)	0.000484 (pH 7.2)	graphite electrode	L‐lactate	graphite rod electrode	2.01 (I_max_) (505 mV)	–	[16a, 38c, 55]
Cellobiose dehydrogenase EC 1.1.99.18	*Phanerochaete chrysosporium* CDH	FAD 106 (−132), haem *b* 190 (130) (pH 3.0 (7.0)	84 (pH 3.0) 262 (pH 7.0)	18 using cellobiose and ubiquinone (pH 3.0)	45.8 (pH 3.0)	1.151 (pH 4.5)	*Pc*CDH/PdNPs–MWCNTs/SPGE	lactose	PcCDH/PdNPs–MWCNTs/SPGE	538 (I_max_) (490 mV)	449 (490 mV)	[56]
	*Trametes villosa* CDH	FAD 81, haem *b* 204 (pH 3.0)	123 (pH 3.0)	23.6 using cellobiose and DCIP (pH 4.5)	–	0.00691 (pH 5.0)	MUDOH‐modified gold electrode	lactose	graphite rod electrode	–	178 (505 mV)	[57]
	*Myriococcum thermophilum* CDH	haem *b* 151 (pH 5.5)	–	19 using cellobiose and DCIP (pH 5.5)	0.13 (pH 5.5)	4.6 (pH 5.5)	gold electrode coated with PDADMAC	lactose; cellobiose; glucose	graphite electrode	lactose 26.3; cellobiose 22.3; glucose 68 (I_max_) (688 mV)	lactose 145; cellobiose 253; glucose 0.18 (688 mV)	[40,58]
	*Corynascus thermophilus* CDH	haem *b* 100 (pH 7.5)	–	39 using DCIP and cellobiose (pH 5.0)	–	21.5 (pH 7.4)	AuNPs/BPDT/AuE	lactose	AuNPs/BPDT/AuE	1045 (I_max_) (449 mV)	275 (449 mV)	[59]
Fructose dehydrogenase EC 1.1.99.11		FAD −26, 3 haems 143, 259, 545 (pH 5.5)	169 (pH 5.5)	95 using fructose and ferricyanide (pH 4.5)	–	580 (pH 5.5)	MWCNT on GCE	D‐fructose	TRGO on GCE	–	145 (605 mV)	[60]
							D‐fructose	MPA modified nanoporous gold electrode	–	37 (355 mV)		
Gluconate 2‐dehydrogenase EC 1.1.99.3		heam *c* 231 (pH 4.5)	–	900 using D‐gluconate and 1,4‐Benzoquinone (pH 4.5)	–	45 (pH 5.0)	carbon paste electrode	D‐gluconate	gold electrodes modified with positively charged thiol	220 (I_50mM_) (599 mV)	–	[18, 62]
Class 2: PQQ‐enzymes carrying a cytochrome												
Pyranose dehydrogenase EC 1.1.2.B5		haem *b* 130 (pH 7.0)	–	53.5 using L‐fucose and cytochrome c (pH 8.5)	–	0.156 (pH 8.5)	glassy carbon electrode	L‐fucose	glassy carbon electrode	128 (I_max_) (505 mV)	–	[20, 63]
Alcohol dehydrogenase EC 1.1.5.5		PQQ 227; 4 haems 265, 428, 431, 496 (pH 4.5)	41 (pH 4.5)	71 using ethanol and DCIP (pH 7.4)	–	0.0624 (pH 7.15)	screen printed carbon electrode	ethanol	screen printed carbon electrode	320 (I_30mM_) (158 mV)	–	[64]
Aldehyde dehydrogenase EC 1.2.5.2				222000 using acetaldehyde and ferricyanide (pH 3.5)	–	0.0276 (pH 7.15)	screen printed carbon electrode	acetaldehyde	screen printed carbon electrode	1.3 (I_30mM_) (158 mV)	–	[64b, 65]
Class 3: Molybdenum cofactor enzymes carrying a cytochrome												
Sulfite dehydrogenase EC 1.8.2.1		Mo^VI/V^ 172, Mo^V/IV^ 31, Fe^III/II^ 242 (pH 8.0)	70 (pH 8.0)	63.5 using sulfite and cytochrome *c* (pH 6.0)	730 (pH 6.0)	0.145 (pH 8.0)	edge‐plane pyrolytic graphite working electrode	sulfite	edge‐plane pyrolytic graphite working electrode	77.8 (I_max_) (200 mV)	–	[24–25, 66]
Sulfite oxidase EC 1.8.3.1		Mo^VI/V^ 131 (−57), Mo^V/IV^ 86 (−233), Fe^III/II^ 90 (51) (pH 6.0; 9.0)	41 (pH 6.0), 108 (pH 9.0)	92.6 using sulfite and cytochrome *c* (pH 8.0)	2–4 (pH 8.0)	17 (pH 7.35)	C8(NH_2_) modified nanostructured Ag electrode	sulfite	platinized glassy carbon	–	382 (705 mV)	[46a, 67]
Class 4: Iron‐sulfur enzymes carrying a cytochrome												
Nitrate reductase EC 1.7.5.1		haem *b*_D_ 14, haem *b*_P_ 118, 3Fe‐4 S 180 (pH 7.0)	62 (pH 7.0)	68 using reduced plumbagin and nitrate (pH 7.0)	9.24 (pH 7.0)			nitrate	pyrolytic graphite “edge” rotating electrode	900 (I_1mM_) (‐400 mV)	–	[31,68]
Designed cytochrome domain fusion enzymes												
fusion enzyme *Af*GDH‐CYT		haem *b* 162 (pH 4.5)	–	780 using glucose and DCIP (pH 6.5)	0.172 (pH 6.0)		MWCNTs on SPCE	glucose	MWCNTs on SPCE	8.5 (I_50mM_) (605 mV)	0.49 (605 mV)	[69]
fusion enzyme *Bc*GDH‐CYT		FAD −345, haem −25 (pH 5.0)	320 (pH 5.0)	1.7 using glucose and DCIP (pH 7.0)	–		glassy carbon electrode	glucose	glassy carbon electrode	20 (I_max_) (205 mV)	–	[70]

The redox potential of the cytochromes and the other cofactors depends on pH, temperature, buffer species and concentration and therefore the values from literature are different to compare. However, the redox potential difference between the cofactors measured at the same conditions is interesting to compare. Especially the flavoenzymes have a wide spread redox potential difference, e. g. *P. chrysosporium* CDH with 84 or 262 mV (pH 3.0 or 7.0, respectively. For PQQ‐ or molybdenum‐dependent, iron‐sulfur cluster enzymes, smaller potential differences between 41 mV and 70 mV were measured. Even the smallest redox potential difference provides a sufficient driving force over the short distances to allow for fast IET. The efficiency of the IET can be estimated by considering the steady‐state turnover number (TN) of the active‐site. An IET faster than the TN makes the catalytic reaction rate limiting and is unlikely to be a criterion for further evolutionary selection. A slow IET rate reduces the catalytic turnover and would be subjected to evolution. The found IET rates (a first order rate typically determined by stopped‐flow spectroscopy) are therefore in a similar range as the TN. For example the IET rate in L‐lactate dehydrogenase is 90 s^−1^, whereas the catalytic TN is 214 s^−1^ (measured with ferricyanide as one‐electron acceptor of the flavin). So the cytochrome‐flavin IET is very efficient and only little rate limiting. In cellobiose dehydrogenase a broad range of IET rates can be found 0.13–45.8 s^−1^), which is due to the different enzyme producers and possible slightly different physiological functions and conditions, but also pH is an important factor and restricts IET in most CDHs to pH values below 7 with maxima between pH 3–5.

Whereas the IET between the two protein redox partners has been evolved and optimized over time, the DET to electrode surfaces likely depends less on the protein, but more on the complementarity of the electrode surface. A suitable environment in regard to hydrophobicity, hydrophilicity, charges, ion species and concentration, etc. has to be provided to optimize DET. Nevertheless, for some enzyme/electrode combinations high DET rates, comparable to the TN and IET rates are reported, e. g. for fructose dehydrogenase, gluconate 2‐dehydrogenase and cellobiose dehydrogenase. For enzymes with no reported DET rates the minimum DET rate based on the enzyme loading and the obtained current was recalculated in Table 2. These rates do of course grossly underestimate the real DET, but still show that only a fraction of the absorbed/immobilized enzymes are in direct communication with the electrode surface.

The efficiency of the whole electron transfer (IET+DET) can be assessed from the reported current densities of the enzyme‐modified electrodes. Specific current densities between 1.3 and 1045 nA mm^−2^ are reported. Especially for enzymes/electrode combinations with a low current density a suboptimal DET can be assumed. However, it has to be taken into account that the actual surface area in contact with the enzyme and the geometric surface area used for the calculation differ greatly. Nanomaterials and chemical modifications of electrode surfaces have been used to increase the available surface for enzyme immobilization and to optimize the DET by improved binding and orientation of the enzyme. However, also simple, unmodified electrodes based on carbon work very well. When used and calibrated as biosensors, the enzyme‐modified electrodes reach sensitivities between 31 and 275 nA mm^−2^ mM^−1^.

## 6 Direct Electrochemistry of Flavocytochromes

DET‐capable enzymes from the classes of flavocytochromes and PQQ‐dependent multi‐cofactor enzymes will be discussed in detail in the following sections, by using the above derived framework of molecular and functional properties to evaluate their performance on electrodes.

### 6.1 Cellobiose Dehydrogenase

Cellobiose dehydrogenase (CDH) is one of the best‐documented DET‐enzymes for application in biosensor and biofuel cells. It is an extracellular flavocytochrome secreted by fungi to obtain and transfer electrons from the oxidation of its natural substrate cellobiose to its physiological electron acceptor lytic polysaccharide monooxygenase.^53^ CDH is thereby involved in the oxidative degradation of cellulose, hemicellulose and chitin. Because of its extracellular destination, CDH is glycosylated, which is necessary to avoid proteolytic degradation and to increase solubility and stability.

The crystal structure of the full‐length CDH was reported in 2015.39b CDHs from two different fungi were structurally resolved and showed two different conformational states. For *Myriococcum thermophilum CDH* the closed‐state with the β‐sandwich cytochrome *b* domain bound to the glucose‐methanol‐choline (GMC)‐oxidoreductase flavodehydrogenase domain was observed (4QI6), whereas for *Neurospora crassa* CDH the open‐state with a swung‐out cytochrome domain was found (4QI7). The structures demonstrate that the cytochrome‐dehydrogenase interaction is not strong and therefore the cytochrome domain is mobile, with a distance of 3.87 nm between both domains in the crystal structure of the open‐state *N. crassa* CDH.

Site‐directed mutagenesis confirmed the interaction site of both domains. The biggest contribution to binding comes from the electrostatic interaction of a haem propionate‐A group interacts with an Arg residue on the dehydrogenase domain on the side of the substrate channel.39b High‐speed atomic force microscopy was used to visualize the dynamic domain motion of *P. chrysosporium* CDH.^54^ An flip‐flop motion was observed involving domain‐domain association in the presence of the substrate cellobiose and subsequent dissociation, whereas the two domains were immobile in the absence of substrate. The elongated conformation of CDH in oxidized state was also confirmed by small‐angle neutron scattering measurement.^71^ These comprehensive structural and functional studies demonstrate the function of CDH's cytochrome domain for DET.

In CDH the IET is considered the rate‐limiting step, especially at less acidic pH (above 5.0).16c,^72^ In *M. thermophilum* CDH the IET rate is especially low, about ∼150 times lower than the TN (pH 5.5 (Table 2). Divalent cations were found to promote the domain interaction and enhance the IET at neutral or alkaline pH, most probably by reducing the electrostatic repulsion between both domains (cytochrome domain pI=4.07, dehydrogenase domain pI=4.89). At a 30 mM CaCl_2_ concentration the IET rate was increased ∼80 fold.58b Hydrogen/deuterium exchange mass spectrometry (HDX‐MS) revealed that the domain interaction was promoted via the neutralization of negative charges by divalent cations at the domain interface.^73^ The conformational rearrangement of CDH's domains in the presence of calcium ions was also observed by small angle X‐ray scattering and small angle neutron scattering.39b,^74^ The effect of divalent cations on some CDHs was first reported by C. Schulz, who observed an enhanced catalytic current of CDH in the presence of calcium ions,^75^ which points towards a limiting IET rate. Weidinger found a calcium‐induced reorientation of CDH on electrodes employing surface‐enhanced vibrational spectroscopy.^40^ The reported DET rate of 4.6 s^−1^ is 35 times higher than IET rate measured by stopped‐flow spectroscopy (Table 2), which confirms that IET is the overall rate‐limiting step in *M. thermophilum* CDH.

Based on its ability to perform DET, CDH has been studied to develop third generation biosensors and enzymatic biofuel cells.16c,16d CDH from Basidiomycota are commonly used for lactose and cellobiose biosensors due to the high substrate specificity.^76^ CDH from certain Ascomycota with IET and DET at neutral pH and a higher catalytic activity towards glucose is applied for glucose biosensors.^77^ Different electrode materials, nanomaterials and functional groups were applied to investigate and optimize the electrode architecture for CDHs, which has been reviewed in detail.16c,^72^,^78^ Later research studied changes in the electrode surface charge in more detail. Three different CDHs have been immobilized onto alkanethiol‐modified gold electrodes.^79^ Positively charged surfaces showed an enhancement on the DET rate. A covalent immobilization method for CDH was developed to immobilize CDH on carbon electrodes via a surface exposed cysteine introduced by genetic engineering.^80^ Different CDH orientations on the electrode were obtained and studied in regard to optimum DET (manuscript in preparation). Similar to the electrodes, also the enzyme has been modified. It has been reported that glycosylation can reduce the electron transfer of oxidoreductases, especially for enzymes with deeply buried prosthetic groups.^81^ The effect of deglycosylation was therefore studied and a ∼3‐fold enhancement of DET was found for deglycosylated CDHs.^82^ Instead of deglycosylating the CDH produced by the yeast *Pichia pastoris* (which is known to overglycosylate and O‐glycosylate recombinant proteins), CDH also can be produced in alternative expression systems.^83^ The filamentous fungi *Trichoderma reesei* turned out to be the best host to produce low‐glycosylated CDH of high uniformity of the glycoform.

### 6.2 Flavocytochrome *b_2_*

Flavocytochrome *b_2_* (Fcb_2_) is an L‐lactate:cytochrome *c* oxidoreductase found in the intermembrane space of yeast mitochondria. Fcb_2_ is homotetrameric enzyme that catalyzes the oxidation of L‐lactate to pyruvate with a subsequent reduction of cytochrome *c*. The first crystal structure of Fcb_2_ was published in 1990.38a Each subunit of Fcb_2_ contains one flavin mononucleotide (FMN) and one haem *b* in two structurally and functionally distinct domains. The two domains are linked by an interdomain “hinge” peptide and there is a C‐terminal “tail” which is involved in the tetramer integrity. Mutational studies of the functional residues in active‐site together with molecular dynamics studies uncovered the substrate oxidation mechanism.^84^ The substrate specificity of Fcb_2_ has been successfully changed toward larger (*S*)‐2‐hydroxy acids by enzyme engineering,39b,^85^ which broadens the biosensing applications of Fcb_2_.

The IET in Fcb_2_ was also studied in detail by stopped‐flow spectroscopy, laser flash photolysis and temperature jump experiments. These confirmed the IET from the fully reduced FMN to haem *b* is several times faster than the catalytic turnover.^86^ However, the second electron transfer from the FMN semiquinone to haem *b* was shown to be much slower and to be the overall rate‐limiting step in the catalytic cycle.38c,^87^ Neither shortening nor extension of the hinge peptide resulted in a dramatic change of the IET.^88^ Based on structural and NMR data, the mobility of the cytochrome domain was proposed to be the key factor controlling IET.^89^ In the Fcb_2_ crystal structure, the cytochrome domain is moved relative to the tetrameric flavodehydrogenase domain core in one out of two subunits.89c A mutational analysis in and around the cytochromes domain interface was analyzed by kinetics, spectroscopic and electrochemical measurements to elucidate the cytochrome domains binding to the dehydrogenase domain and to cytochrome *c* as electron acceptor.^55^ The interaction sites on the cytochrome domain for both the flavodehdrogenase domain and cytochrome *c* overlap, which demonstrates the cytochrome domain's mobility, which has to move away from the flavodehydrogenase domain for the interaction with cytochrome *c*.

Both DET and MET sensors based on Fcb_2_ were developed for L‐lactate determination in the 1980s.^2^,^90^ However, because of the low obtained DET current, following work focused on a Fcb_2_ overproducing yeast, *Hansenula polymorpha*, which was used as biological recognition element in amperometric L‐lactate biosensors and DET was achieved on different electrode materials.16b The proposed electron transfer pathway consists of three redox proteins, Fcb_2_, cytochrome *c* and Complex‐IV in a cascade. The sensitivity of the best obtained graphite electrode is 250 nA mM^−1^. Also different redox mediators were tested in combination with the yeast cells to improve the performance.^91^ as well as gold nanoparticles.^92^ Also a different yeast, *Saccharomyces cerevisiae*, was employed to create a yeast‐modified, mediated amperometric biosensor for lactic acid detection.^93^ Besides it obvious application for the determination of physiological lactate concentrations, an application for the quality control in wine is also considered.^94^

## 7 Electrocatalytic Applications of PQQ‐Dependent Enzymes with a Cytochrome

### 7.1 PQQ‐Dependent Pyranose Dehydrogenase from Basidiomycota

The first PQQ‐dependent enzymes in an eukaryote was reported in 2014 by Nakamura. It is a PQQ‐dependent pyranose dehydrogenase (PDH) from the basidiomycete *Coprinopsis cinerea* carrying a cytochrome *b* domain.^95^ The amino acid sequence of PDH indicates three domains of the extracellular enzyme: an N‐terminal cytochrome domain, a central catalytic PQQ‐dehydrogenase domain and a C‐terminal family 1 carbohydrate binding module (CBM1). The haem *b* containing cytochrome domain has a β‐sandwich fold and is homologous to the cytochrome domain of cellobiose dehydrogenase. Like CDH, PDH can also function as electron donor of LPMO.^96^ The roles of the individual PDH domains were determined by enzyme variants consisting of 1, 2 or 3 domains. The cytochrome domain is needed for the electron transfer from the PQQ cofactor in the dehydrogenase domain to the LPMO, which activates the LPMO and is essential for reoxidizing PDH. Interestingly, the CBM1 domain was reported to enhance the electron transfer efficiency of the PDH‐LPMO systems but the mechanism is not clear.^96^

DET from PDH to glassy carbon electrodes has been observed using L‐fucose as a substrate.^20^ The formal potential of the haem *b* in the cytochrome domain was determined by cyclic voltammetry to be 130 mV vs. SHE,^63^ it is generally very similar to the biophysical properties of the CDH cytochrome domain. The PQQ cytochrome domain can also interact with cytochrome *c* or electrodes as terminal electron acceptors. Bioelectrocatalysis of PDH demonstrated that the measured pH profile of the biocatalytic current was similar to the pH profile of cytochrome *c*. The optimal pH values is 8.5.^20^ These preliminary data are promising and show that PDH can be used in DET‐based pyranose biosensors and biofuel cell anodes.

### 7.2 PQQ‐Dependent Dehydrogenase from Acetic Acid Bacteria

For PQQ‐dependent alcohol dehydrogenases (ADH) and aldehyde dehydrogenases (AldDH) from acetic acid bacteria, mainly *Gluconobacter* and *Acetobacter*, DET has been demonstrated almost 2 decades ago.^19^ Membrane‐associated ADHs and AldDHs consist of a PQQ subunit, a haem *b/c*‐containing subunit and additionally a multihaem *c*‐containing subunit as well as a PQQ subunit. The intramolecular electron transfer route was proposed after determining the redox potentials of all four haem *c* cofactors.64c Based on the crystal structure of two different ADHs, the lengths of the linker regions connecting the PQQ and the cytochrome domains are different, leading to a significant difference in the orientation of the cytochrome domain with respect to the PQQ domain.45b

The DET of ADH and AldDH was first observed on screen printed carbon electrode. The achieved current densities with 30 mM ethanol/acetaldehyde was 320 or 1.3 nA mm^−2^, respectively.64b Later on, the bioelectrooxidation of ethanol using these two enzymes was explored further for biofuel cell application. A catalytic current of 168 nA mm^−2^ was achieved based on the DET of both enzymes.^21^ In order to investigate the best orientation of the large and complex multi‐subunit enzyme AldDH, it was site‐specifically immobilized on electrodes with His_6_‐tags. The most favorable orientation was found for the variant with the haem *c* in closest proximity to the electrode.^97^

Other isolated PQQ‐dependent enzymes from acetic acid bacteria are a lactate dehydrogenase (LDH), which showed DET on both gold and carbon electrode surfaces with maximum current density of 360 nA mm^−2^ at a 50 mM lactate concentration (pH 7.15).22a Another new discovered pyruvate dehydrogenase from *Gluconobacter* showed a low substrate specificity, which is advantageous for the deep oxidation of the pyruvate (completely oxidized to carbon dioxide) in biofuel cell anodes,22b which achieved a maximum current density of 319 nA mm^−2^ at a 25 mM pyruvate concentration (pH 7.15).

## 8 Generating DET Enzymes by Fusion of a Cytochrome Domain

The search for specific biosensor catalysts with DET properties has driven research to generate DET enzymes based on FAD‐dependent glucose dehydrogenase (GDH) for glucose biosensors. GDH is a thermostable, oxygen insensitive redox enzyme widely used in second generation biosensors. Since the FAD cofactor of the enzyme is deeply buried within the protein, it is almost impossible to achieve efficient DET. Fusion of GDH with a cytochrome domain was successfully introduced as a way of creating new multi‐cofactor enzymes capable of DET.^70^,^98^

**Figure 8:**
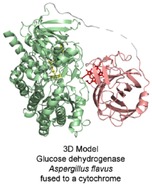
The fusion enzyme of the *P. chrysosporium* CDH cytochrome *b* (red) and *A. flavus* glucose dehydrogenase (green) in a schematic representation. The N‐terminally fused cytochrome *b* is shown in a closed conformation; the protein linker region (grey dashes) is undefined.

To that purpose, the group of K. Sode fused the β‐sandwich cytochrome *b* domain of CDH from *P. chrysosporium* N‐terminally to glucose dehydrogenase from *Aspergillus flavus* (Figure 8).69c The designed multi‐cofactor GDH exhibited DET on carbon electrodes. The IET between the FAD and the haem *b* cofactors based on spectroscopic measurements is reported to be 0.17 s^−1^ (pH 6.0, a value comparable to *M. thermophilum* CDH. The IET was modulated by pH, but only slightly (2‐fold) by the presence of the divalent Ca^2+^ ions.69c Also the apparent K_M_ value of the electrode immobilized enzyme for glucose was determined. The K_M_ of the heterogenized enzyme is 8.6 mM and 6‐times lower than the K_M_ obtained in solution (53 mM), which indicates that the catalytic reaction is limited by either IET or DET. Since the cytochrome domain of *P. chrysosporium* CDH exhibits a high DET, the IET between the cofactors is rate limiting. The measured specific current density at a 5 mM glucose concentration and 605 mV vs. SHE was ∼4.5 nA mm^−2^.

**Figure 9:**
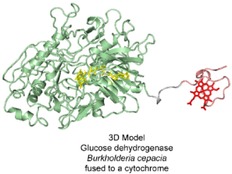
The fusion enzyme of the MamP cytochrome *c* (red) and *B. cepacia* glucose dehydrogenase (green) in a schematic representation. The C‐terminally fused cytochrome *c* is shown in the open conformation, the protein linker region (grey line) is undefined.

Another study by the group of L. Alfonta replaced the large, 43 kDa multi‐haem cytochrome c subunit of *Burkholderia cepacia* glucose dehydrogenase by a much smaller, cytochrome *c* domain from the magnetochrome‐containing protein MamP^99^ which consists of only 23 amino acids and has a low redox potential of −89 mV vs. SHE.^100^ The cytochrome domain was C‐terminally attached to the flavodehydrogenase domain to achieve direct electron transfer (Figure 9).^70^ The constructed fusion enzyme exhibits an about 3‐times higher catalytic activity than the wild‐type GDH. This more active preparation of the fusion enzyme showed a 5‐times higher catalytic current at a 5 mM glucose concentration (20 nA mm^2^) than the wild‐type GDH (4 nA mm^2^) at a lower onset potential (∼50 mV vs. SHE), which improves the glucose sensing properties of the enzyme. The use of a mobile heptapeptide linker (GSGYGSG) and the choice of a small cytochrome domain improved the overall electron transfer to the electrode, but it was not elucidated if IET or DET was improved.

The function of *B. cepacia* GDH's multi‐haem cytochrome *c* subunit was investigated by a truncation experiment.^101^ Based on modelling the structure on homologous cytochromes the haem 3 and its surrounding protein region was identified to contact the FAD and it was produced as a truncated subunit. The GDH‐truncated cytochrome complex exhibited only a slow IET and DET (∼4 nA mm^2^ at a 5 mM glucose concentration) on a thioalkane modified gold electrode.

The GDH from *A. flavus* has a much higher catalytic TN for glucose (780 s^−1^) than GDH from *B. cepacia* (1.7 s^−1^) or CDH from *C. thermophilus* (39 s^−1^). This high glucose activity is a good basis for a DET biocatalyst and keeps the FAD cofactor in its reduced form which enhances the probability of an IET event. On the other hand, reduced FADH_2_ is susceptible to the futile generation of H_2_O_2_, if the electrons are not removed quickly from the cofactor, which would compromise the stability of the biosensor catalyst. The abstraction of the first electron from FADH_2_, leading to the FAD semiquinone suppresses the generation of H_2_O_2_. Besides efficient electron transfer to the electrode, this is another argument for a fast IET by selecting the most suitable cytochrome domain.

The catalytic rate of the *A. flavus* fusion enzyme is limited by the IET, which was reported to be 0.172 s^−1^. For the *B. cepacia* fusion enzymes no IET rate was reported, but by considering the specific current densities for both enzymes, which are 20 and 8.5 nA mm^−2^, respectively (Table 2), a strongly limiting IET is highly likely. However, these are promising current densities for a proof‐of‐concept. Compared to the best performing CDH‐based glucose biosensor electrode with 1045 nA mm^−2^ the fusion enzymes have already a good starting performance, which can be further increased by protein engineering and modification of the electrode architecture. An increase of the IET rate would be very beneficial and can be achieved by protein engineering of the domain interface. Different strategies for an improved IET can be envisaged: (1) It is known from CDH that charge repulsion plays a big role in the interaction of the flavodehydrogenase domain and the cytochrome domain. Studies of the pH‐dependent domain interaction as already performed69c can be used to investigate the influence of charged amino acids at the interface. (2) Another important aspect is surface complementarity and the edge‐to‐edge distance of the cofactors. Although larger electron transfer distances between cofactors are known than those summarized in Table 1, the edge‐to‐edge distance in enzymes with a mobile cytochrome domain rarely exceeds 1 nm. A bad complementarity of the domains in fusion enzymes can easily result in a larger distance and a deteriorated IET. (3) Similarly, the preference of the fusion enzyme for the closed conformation is important to achieve efficient IET. Here an optimum has to be found, since DET depends (beside a suitable electrode architecture) on the open conformation of the cytochrome domain and an orientation on the electrode that gives the cytochrome domain maximum freedom to contact the catalytic domain as well as the electrode.

These three factors demonstrate that the selection of the cytochrome for a fusion enzyme is a difficult choice and that existing cytochromes need adaption to complement and interact with the catalytic domain, which currently restricts the generation of fusion enzymes. Although the mobility of the cytochrome is important to shuttle electrons between the enzyme and the electrode, the rate limiting step will be in most cases the correct, IET‐competent association of the domains. Protein‐protein docking and molecular dynamics (MD) simulations are the methods of choice to engineer compatible interaction surfaces that allow a fast association of the cytochrome to the catalytically active domain, but also allow a fast dissociation to transfer the electron to the electrode.

## 9 Conclusions

Multi‐cofactor enzymes featuring a cytochrome domain or a cytochrome subunit are engaged in diverse, mostly oxidative, reactions and use other redox proteins or redox enzymes as electron acceptors/donors. All found and summarized enzymes in this review use either a *b*‐type cytochrome or a *c*‐type cytochrome for the electron transfer. The selection of the cytochromes might reflect the necessary redox potential (*b*‐type cytochromes 34–264 mV vs. SHE, *c*‐type cytochromes 220–494 mV vs. SHE). In reductases the redox potential needs to be lower than the active‐site cofactor, which is achieved by a low haem redox potential between −238–20 mV vs. SHE). The smallest found redox potential difference between an active‐site cofactor and a cytochrome is 41 mV, but typically it is higher than 80 mV.

The cellular location of an enzyme determines no prevalence of a cytochrome type and a similar number of cytochrome *b* and cytochrome *c* domains or subunits can be found in matrix‐associated and soluble intra‐ or extracellular enzymes. The size and fold of the cytochromes differ, but has no obvious effect on IET or DET rates. No tendency of smaller cytochromes for a faster IET or DET has been found, but for many enzymes a more detailed characterization of these rates is required. For some of the classified multi‐cofactor enzymes a DET to electrodes has not yet been demonstrated. The obvious reason is that some of these enzymes are not suitable, but it is also obvious that the detection of DET also depends on a pure and active enzyme preparation, a suitable electrode, surface structure and chemical substitution. By optimizing the electrode architecture new DET‐enzymes can be discovered and the performance of enzymes with a demonstrated DET can be increased.

The fusion of cytochromes to catalytically interesting enzymes is a promising strategy to generate DET‐enzymes for new substrates/analytes. Initial research has proven the validity of this concept and it will be interesting to see the rational design of fusion enzymes tackling the need for DET‐capable biosensor catalysts. The optimization of the cytochrome domain's redox potential to be sufficient for fast IET, but not wasting to much potential difference, the obtained voltage under load of biofuel cells can be increased and biosensors with a lower polarization potential might also suffer less from interferences of electroactive physiological or pharmaceutical compounds such as ascorbate or paracetamol.

Which design guidelines for the rational engineering of efficient DET‐enzymes can be derived from the existing data? The protein interface of catalytic domain and cytochrome domain has to be sterically and electrostatically complementary. The edge‐to‐edge distance of the cofactors should be as close as possible in the closed conformation. In case of a mobile cytochrome domain a balance between the factors supporting a closed conformation and the open conformation is necessary to facilitate IET as well as DET. Also the length and composition of the protein linker between the domains is important to support, but also restrict the cytochrome mobility. Future studies in this direction will certainly improve our knowledge on how to rationally design DET‐enzymes, but careful measured data are necessary to evaluate the results.

## Conflict of interest

The authors declare no conflict of interest.

## Biographical Information

*Su Ma received her PhD degree in 2013 from Ocean University of China. In 2014, she joined the Biocatalysis and Biosensing Laboratory (BBL) at BOKU – University of Natural Recourse and Life Science, Vienna (Austria) as a Marie Curie research fellow. She continued postdoctoral research for one year in an ERC Consolidator Grant Project and is now a junior PI managing different projects of the BBL in the field of protein engineering and bioelectrochemistry*.

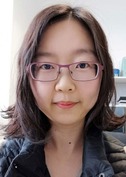

## Biographical Information

*Roland Ludwig received his PhD in biochemistry from BOKU – University of Natural Resources and Life Sciences, Vienna (Austria) in 2004. He worked as a postdoctoral researcher at the Research Centre Applied Biocatalysis, Graz (Austria) and at Lund University (Sweden). In 2011, he started the Biocatalysis and Biosensing Laboratory at BOKU where he studies enzymes by protein engineering, characterization and applications in biocatalytic processes and biosensors*.

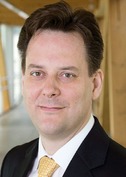

## Supporting information

As a service to our authors and readers, this journal provides supporting information supplied by the authors. Such materials are peer reviewed and may be re‐organized for online delivery, but are not copy‐edited or typeset. Technical support issues arising from supporting information (other than missing files) should be addressed to the authors.

Supplementary
