# Supplementary material for: Direct Electron Transfer of Enzymes Facilitated by Cytochromes
Source: ChemElectroChem. 2018 Dec 13;6(4):958–75. doi: 10.1002/celc.201801256 (PMC6472588; doi:10.1002/celc.201801256)
Supplement: Supplementary file 1 — Supplementary [file CELC-6-958-s001.pdf]

Supporting Information

© Copyright Wiley-VCH Verlag GmbH & Co. KGaA, 69451 Weinheim, 2019

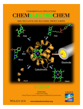

Direct Electron Transfer of Enzymes Facilitated by Cytochromes

Su Ma and Roland Ludwig*© 2018 The Authors. Published by Wiley-VCH Verlag GmbH & Co. KGaA. This is an open access article under the terms of the Creative Commons Attribution License, which permits use, distribution and reproduction in any medium, provided the original work is properly cited.An invited contribution to a Special Collection dedicated to Bioelectrochemistry

Enzyme name	Enzyme EC	catalyzed reaction	cellular location	structural organision	cytochrome mobility	cofactors/haem type	redox partner of CYT
Class 1: Flavoenzymes carrying a cytochrome							
L-lactate dehydrogenase	EC 1.1.2.3	(S)-lactate + 2 ferricytochrome c = pyruvate + 2 ferrocytochrome c + 2 H+	soluble	homotetramer	mobile	FMN, haem b	cytochrome c
cellobiose dehydrogenase	EC 1.1.99.18	cellobiose + acceptor = cellobiono-1,5-lactone + reduced acceptor	soluble	monomer	mobile	FAD, haem b	cytochrome c, LPMO
nitric oxide dioxygenase	EC 1.14.12.17	2 nitric oxide + 2 O2 + NAD(P)H = 2 nitrate + NAD(P)+ + H+	soluble	monomer	mobile	FAD, haem b	O2
gluconate 2-dehydrogenase	EC 1.1.99.3	D-gluconate + acceptor = 2-dehydro-D-gluconate + reduced acceptor	membrane bound	heterotrimer	fixed	FAD, haem c	acceptor, Hydrogen-acceptor
spermidine dehydrogenase	EC 1.5.99.6	spermidine + acceptor + H2O = propane-1,3-diamine + 4-aminobutanal + reduced acceptor	membrane bound	monomer	mobile	FAD, haem c	Ferricyanide, 2,6-dichloroindophenol and cytochrome c can act as acceptor
4-methylphenol dehydrogenase	EC 1.17.99.1	4-methylphenol + 2 acceptor + H2O = 4-hydroxybenzaldehyde + 2 reduced acceptor	membrane bound	heterodimer	fixed	FAD, heme c	Phenazine methosulfate
sulfide-cytochrome-c reductase	EC 1.8.2.3	hydrogen sulfide + 2 ferricytochrome c = sulfur + 2 ferrocytochrome c + 2 H+	membrane bound	heterodimer	fixed	FAD, haem c	flavocytochrome c
NAD(P)H oxidase	EC 1.6.3.1	NAD(P)H + H+ + O2 = NAD(P)+ + H2O2	membrane bound	homodimer	mobile	FAD, 2 haem b	O2
fructose 5-dehydrogenase	EC 1.1.99.11	D-fructose + acceptor = 5-dehydro-D-fructose + reduced acceptor	membrane bound	heterotrimer	fixed	FAD; 3 haem c	2,6-Dichloroindophenol
fumarate reductase	EC 1.3.1.6	succinate + NAD+ = fumarate + NADH + H+	soluble	monomer	mobile	FAD, 4 haem c	NADH
Class 2: PQQ-enzymes carrying a cytochrome							
PQQ-dependent pyranose dehydrogenase	EC 1.1.2.B5	a D-pyranose + a ferricytochrome c = a dehydro-D-pyranose + a ferrocytochrome c + 2 H+	soluble	monomer	mobile	PQQ, haem b	cytochrome c, LPMO
polyvinyl alcohol dehydrogenase	EC 1.1.2.6	polyvinyl alcohol + ferricytochrome c = oxidized polyvinyl alcohol + ferrocytochrome c + H+	soluble	monomer	mobile	PQQ, haem c	cytochrome c
1-butanol dehydrogenase	EC 1.1.2.9	Butan-1-ol + 2 ferricytochrome c = butanal + 2 ferrocytochrome c + 2 H(+)	soluble	monomer	mobile	PQQ, haem c	cytochrome c
lactate dehydrogenase	EC 1.1.5.B3	L-lactate + quinone = pyruvate + quinol	soluble	monomer	mobile	PQQ, haem c	quinone
lupanine 17-hydroxylase	EC 1.17.2.2	lupanine + 2 ferricytochrome c + H2O = 17-hydroxylupanine + 2 ferrocytochrome c + 2 H+	soluble	monomer	mobile	PQQ, haem c	cytochrome c
alcohol dehydrogenase	EC 1.1.9.1	A primary alcohol + azurin = an aldehyde + reduced azurin	soluble	monomer	mobile	PQQ, haem c	azurin
alcohol dehydrogenase	EC 1.1.5.5	ethanol + ubiquinone = acetaldehyde + ubiquinol	membrane bound	heterodimer	fixed	PQQ, 4 haem c	membrane ubiquinone
aldehyde dehydrogenase	EC 1.2.5.2	an aldehyde + a quinone + H2O = a carboxylate + a quinol	membrane bound	heterodimer	fixed	PQQ, haem b, 3 haem c	membrane quinone pool; 2,6-dichlorophenolindophenol
Class 3: Molybdenum cofactor enzymes carrying a cytochrome							
sulfite oxidase	EC 1.8.3.1	sulfite + O2 + H2O = sulfate + H2O2	soluble	homodimer	mobile	molybdenum, haem b	O2

sulfite dehydrogenase	EC 1.8.2.1	sulfite + 2 ferricytochrome c + H2O = sulfate + 2 ferrocytochrome c + 2 H+	membrane bound	heterodimer	fixed	molybdenum, haem c	cytochrome c-551

Class 4: Iron-sulfur enzymes carrying a cytochrome

succinate dehydrogenase	EC 1.3.5.1	succinate + a quinone = fumarate + a quinol	membrane bound	heterotrimer	fixed	FAD, Fe-S cluster, haem b	quinone
fumarate reductase	EC 1.3.5.4	succinate + a quinone = fumarate + a quinol	membrane bound	heterotrimer	fixed	FAD, Fe-S cluster, 2 haem b	menaquinol and rhodoquinol
nitrate reductase	EC 1.7.5.1	nitrate + a quinol = nitrite + a quinone + H2O	membrane bound	heterotrimer	fixed	molybdenum, Fe-S cluster, haem b	quinone
dimethyl sulfide:cytochrome c2 reductase	EC 1.8.2.4	dimethyl sulfide + 2 ferricytochrome c2 + H2O = dimethyl sulfoxide + 2 ferrocytochrome c2 + 2 H+	membrane bound	heterotrimer	fixed	molybdenum, heme b, Fe-S cluster	cytochrome c2
ethylbenzene hydroxylase	EC 1.17.99.2	ethylbenzene + H2O + acceptor = (S)-1-phenylethanol + reduced acceptor	membrane bound	heterotrimer	fixed	molybdenum, Fe-S cluster, haem b	p-benzoquinone or ferrocenium
chlorate reductase	EC 1.97.1.1	AH2 + chlorate = A + H2O + chlorite	soluble	heterotrimer	fixed	molybdenum, Fe-S cluster, haem b	Flavins or benzylviologen
selenate reductase	EC 1.97.1.9	selenite + H2O + acceptor = selenate + reduced acceptor	soluble	heterotrimer	fixed	molybdenum, haem b, Fe-S cluster	
CoB-CoM heterodisulfide reductase	EC 1.8.98.1	CoB + CoM + methanophenazine = CoM-S-S-CoB + dihydromethanophenazine	membrane bound	heterotdimer	fixed	2 haem b, 2 Fe-S cluster	
hydrogen:quinone oxidoreductase	EC 1.12.5.1	H2 + menaquinone = menaquinol	membrane bound	heterotdimer	fixed	nickel, Fe-S cluster and haem b	water-soluble quinones (e.g. 2,3-dimethylnaphth oquinone) or viologen dyes
nitrate reductase	EC 1.9.6.1	2 ferrocytochrome + 2 H+ + nitrate = 2 ferricytochrome + nitrite	soluble	heterodimer	fixed	molybdenum, Fe-S cluster, 2 haem c	cytochrome
nitrite reductase	EC 1.7.1.15	NH3 + 3 NAD+ + 2 H2O = nitrite + 3 NADH + 5 H+	soluble	homodimer	mobile	FAD, Fe-S cluster, siroheme	NADH
assimilatory sulfite reductase (NADPH)	EC 1.8.1.2	hydrogen sulfide + 3 NADP+ + 3 H2O = sulfite + 3 NADPH + 3 H+	membrane bound	heterotetramer	fixed	sirohaem, Fe-S cluster, FAD, FMN	NADPH

Class 5: Multihaem enzymes carrying a cytochrome

nitrite reductase (NO-forming)	EC 1.7.2.1	nitric oxide + H2O + ferricytochrome c = nitrite + ferrocytochrome c + 2 H+	soluble	monomer	mobile	haem c, haem d1	cytochrome c550, c551 azurin and pseudoazurin
nitrite reductase (cytochrome; ammonia-forming)	EC 1.7.2.2	NH3 + 2 H2O + 6 ferricytochrome c = nitrite + 6 ferrocytochrome c + 7 H+	membrane bound	homodimer	fixed	5 haem c	cytochrome
trimethylamine-N-oxide reductase (cytochrome c)	EC 1.7.2.3	NADH + H+ + trimethylamine N-oxide = NAD+ + trimethylamine + H2O	soluble	monomer	fixed	5 haem c	cytochrome c

nitric oxide reductase (cytochrome c)	EC 1.7.2.5	nitrous oxide + 2 ferricytochrome c + H2O = 2 nitric oxide + 2 ferrocytochrome c + 2 H+	membrane bound	heterodimer	fixed	non-haem iron centre, haem b3, haem c, haem b	cytochrome c
hydroxylamine dehydrogenase	EC 1.7.2.6	hydroxylamine + 3 ferricytochrome c = nitric oxide + 3 ferrocytochrome c + 3 H+; hydroxylamine + H2O + 4 ferricytochrome c = nitrite + 4 ferrocytochrome c + 5 H+	soluble	homotrimer	fixed	7 haem c	cytochrome c
hydrazine synthase	EC 1.7.2.7	hydrazine + H2O + 3 ferricytochrome c = nitric oxide + ammonium + 3 ferrocytochrome c	membrane bound	heterotrimer	fixed	2 haem c	cytochrome c
hydrazine dehydrogenase	EC 1.7.2.8	hydrazine + 4 ferricytochrome c = N2 + 4 ferrocytochrome c; hydroxylamine + NH3 + acceptor = N2 + H2O + reduced acceptor	soluble	homodimer or homotrimer	fixed	8 haem c	quinone
